# Light driven polymer thin films as flying robotic chips in the sky

**DOI:** 10.1039/d5lc00900f

**Published:** 2026-01-07

**Authors:** Jianfeng Yang, Hao Zeng

**Affiliations:** a Light Robots, Faculty of Engineering and Natural Sciences, Tampere University P.O. Box 541 FI-33101 Tampere Finland jianfeng.yang@tuni.fi hao.zeng@tuni.fi

## Abstract

Beyond conventional locomotion methods such as walking and swimming, flying remains an unconquered frontier for responsive materials. Current aerial vehicles, which rely on electric motors or actuators, face challenges in terms of power density and miniaturization. Nature, however, offers inspiration: wind-assisted passive flight mechanisms seen in seeds provide highly energy-efficient models for microroboticists. This review highlights interdisciplinary efforts aimed at harnessing responsive thin films to create aerial systems with mid-air controllability and robotic capabilities. We explore biological designs for wind-dispersed flyers, the underlying flight mechanisms, and materials for shape-morphing and robotic flight control. Additionally, we examine the potential for onboard sensing and discuss the risks and challenges facing this emerging research field.

## Abstract 2

We review interdisciplinary efforts to create miniature aerial systems with mid-air controllability and robotic capabilities using responsive thin-film materials.

## Graphical abstract

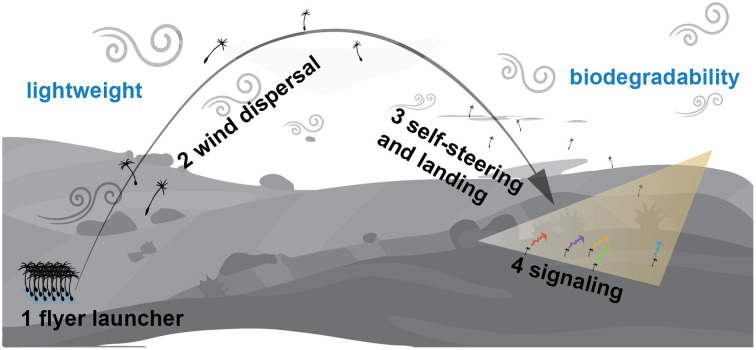

## 1. Introduction

Flight has fascinated humanity for millennia. The dream of soaring through the air like birds or insects has fuelled relentless innovation, culminating in the Wright brothers' historic powered flight on December 17, 1903. Over the past century, this dream has materialized into vast aerospace achievements – from supersonic jets to colossal airliners like the Airbus A380.^1^ Yet, as aviation matured, attention shifted from sheer scale and speed to miniaturization and manoeuvrability, unlocking new realms of possibility in aerial robotics. The 1990s marked a pivotal moment when the U.S. Defense Advanced Research Projects Agency (DARPA) introduced the concept of bioinspired micro aerial vehicles (BMAVs).^2^ This initiative fused biology with engineering, giving birth to a vibrant interdisciplinary field centered on developing micro aerial vehicles (MAVs) at insect scale or smaller.^3–6^ These devices, typically weighing a few grams or less, harness novel materials and designs to replicate the remarkable agility, resilience, and aerodynamic efficiency of natural flyers.^7,8^ MAVs hold transformative promise across diverse applications – environmental monitoring in fragile ecosystems, precision agriculture targeting crop health, urban search-and-rescue in confined rubble, and disaster response in hazardous conditions inaccessible to larger drones.^3,4^

Conventional electromechanical actuators, particularly electric motors, face fundamental miniaturization limits as system mass approaches the sub-gram range.^9,10^ Key physical constraints include declining current density due to increased electrical resistance, reduced electromagnetic force output from smaller motor geometries, and the necessity of high gear ratios that introduce mechanical inefficiencies and energy loss.^11,12^ Consequently, traditional motors become impractical or ineffective for ultra-small MAVs, necessitating alternative actuation strategies.^13^ Inspired by nature's solutions, researchers have increasingly explored artificial muscles – materials and devices capable of reversible contraction, expansion, or twisting in response to external stimuli such as electrical fields, heat, or light.^14–18^ Dielectric elastomer actuators (DEAs) have emerged as a particularly promising class.^19–22^ DEAs enable MAVs to perform agile maneuvers, absorb collisions without damage, and tolerate partial structural failures – qualities essential for robust flight in unstructured and unpredictable environments.^19,23^ Recent demonstrations have shown flying robots with collision resilience and dynamic stability rivaling insects, suggesting that soft actuation can overcome many traditional limitations of rigid mechanical systems. However, realizing fully autonomous MAVs powered by DEAs remains challenging. The high voltages required (typically 500 to 5000 volts) complicate onboard power supply and control integration, introducing concerns regarding energy efficiency, long-term reliability, and safety.^24^

Against this backdrop, nature offers alternative paradigms of microscale flight that operate with astonishing energy efficiency and robustness.^25^ Wind-dispersed seeds, such as those of maple and dandelion species, achieve long-range passive flight without any active propulsion.^26^ Their dispersal relies solely on intricate aerodynamic interactions with ambient airflow, enabling them to travel kilometers on wind currents. These botanical microflyers embody elegant principles of passive stability, energy harvesting from environmental flow, and minimal structural complexity – attributes highly desirable for MAV design.

**Fig. 1:**
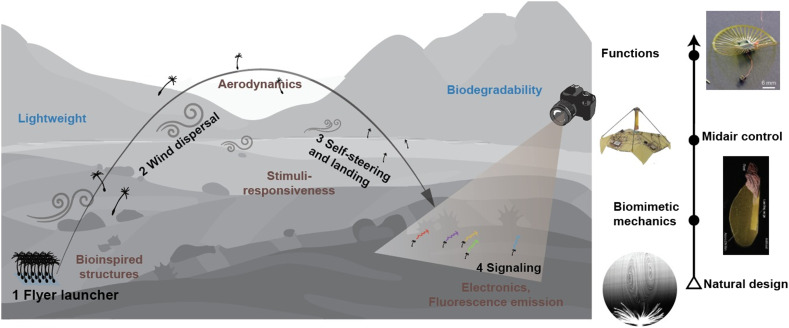
Roadmap for next-generation drones in environmental monitoring. The left panel illustrates the strategies to be implemented along this trajectory, while the right panel traces the design pathway—progressing from bio-inspired concepts and mimicry of natural mechanisms to controlled aerial locomotion and, ultimately, multifunctional system integration. For the right panel, from bottom to top, reproduced from ref. 29 with permission from Springer Nature; copyright 2018; reproduced from ref. 38 with permission from Springer Nature, copyright 2024; reproduced from ref. 39 with permission from AAAS, copyright 2023; reproduced from ref. 31 with permission from Springer Nature.

In recent years, a new roadmap for wind-dispersed flying robots and sensors has gradually emerged.^27,28^Fig. 1 illustrates the working principle of this new generation of flyers proposed, with many intermediate steps already preliminarily demonstrated by several research groups.^29–35^ This roadmap envisions future drones as lightweight, biodegradable polymer structures with bioinspired functions that can disperse over long distances by wind and autonomously decide when to navigate and land based on local environmental conditions. Upon landing, the changes in fluorescence emission or colour can then be used to assess local factors such as humidity, light, and temperature through simple photography or laser spectroscopy.^32,36,37^

Pursuing the goals, researchers have begun to design lightweight structures that not only mimic natural structure but also capture the aerodynamic principles enabling long-distance airborne stability.^29,38^ The integration of stimuli-responsive materials allows these devices to adapt their descent dynamically, providing self-steering and landing in response to environmental cues. Moving beyond passive dispersal, systems are envisioned to host functional payloads – ranging from microelectronics to luminescent materials – for sensing and signal transmission.^39,40^ To minimize ecological impact, the use of biodegradable substrates^37^ has become a central design criterion. Together, these developments across disciplinary boundaries synergize along the line toward bioinspired, eco-friendly aerial microsensors that couple the efficiency of natural dispersal mechanism with engineered material intelligence (left, Fig. 1).

In this review, the “flight” is referred to the controlled or directed motion through air at small scales, where movement is strongly affected by the fluid dynamics and viscous forces. In this kind of flight, conventional aerodynamic mechanisms used in macroscopic aircraft-such as lift generated by stable fixed-wings cease to be effective. We explore how natural flyers inspire emerging strategies for miniature aerial robotics. We begin by surveying biological examples of aerial dispersal and locomotion, emphasizing natural structures that enable energy-efficient flight. We then examine the fluid dynamics underlying passive flight at small scales, with particular attention to unsteady aerodynamics and vortex generation. Recent advances in stimuli-responsive materials are discussed for their roles in enabling shape morphing and sensing in untethered systems. Finally, we highlight key design and control strategies demonstrated in thin-film robotic flyers developed to date.

## 2. Natural examples for flyer design

Plants mastered the art and science of aviation long before Orville and Wilbur Wright propelled their frail craft into the air.^41^ Indeed, passive flight has long been perfected in the botanical world, where it is most strikingly observed in the wind-dispersed seeds of plants. These seeds rely entirely on environmental forces – such as airflow and air pressure differentials – to sustain prolonged airborne transport, navigating the skies without any active propulsion or onboard energy source.^25^

Wind-dispersed diaspores can be broadly categorized into two functional types: winged seeds and tufted, pappus-bearing seeds. Each of these adopts a distinct aerodynamic strategy finely tuned by evolution. Winged seeds typically exploit one of three flight modes, *i.e.*, gliding, tumbling, and autorotation.^42^ These modes are governed by subtle shifts in the seed's centre of mass and by the interaction between the seed body and its extended planar wing structures, which together generate aerodynamic lift. In contrast, seeds equipped with a pappus (*e.g.*, those of the dandelion) utilize a parachute-like architecture to maximize the air drag to induce a slow, stable descent.^43^

### 2.1 Winged seeds

**Fig. 2:**
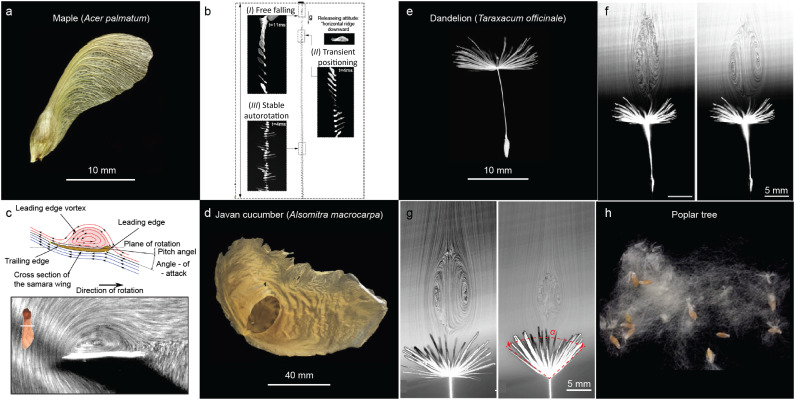
Wind-assisted seeds in nature. a) Photographs of a maple samara. b) Representative descent trajectory of a maple samara in still air, reproduced from ref. 42 with permission from APS, copyright 2022. c) Top: Schematic of sectional streamlines showing the formation of the leading-edge vortex (LEV) on a maple samara wing, reproduced from ref. 51 with permission from MDPI, copyright 2023. Bottom: Experimental visualization of LEV streamlines, reproduced from ref. 53 with permission from AAAS, copyright 2009. d) Photographs of a Javan cucumber seed. e) Photographs of a dandelion diaspore. a, d and e are reproduced from ref. 26 with permission from Elsevier Inc., copyright 2022. f) Steady-state wake patterns formed downstream of a dandelion diaspore under two flow conditions: left, at terminal descent velocity; right, at 60% of terminal velocity, reproduced from ref. 29 with permission from Springer Nature, copyright 2018. g) Flow visualization of a dandelion diaspore with an open pappus (left) and a partially closed pappus (right), reproduced from ref. 70 with permission from *eLife*, copyright 2022. h) Photographs of a cluster of poplar tree seeds.

Winged seeds represent a remarkable evolutionary solution to maximize their horizontal travel distance from the parent tree without the need for any metabolic energy input. Among them, the maple samara is perhaps the most emblematic, exhibiting a finely tuned capacity for autorotation that allows it to descend slowly and stably through the air^42^ (Fig. 2a). Despite the considerable morphological diversity across plant species, winged seeds share surprisingly conserved structural and aerodynamic features that underpin their flight capabilities.

From a structural perspective, maple samaras can be decomposed into several distinct functional regions: a thin planar wing reinforced with longitudinal veins for flexural rigidity, a thickened leading edge that improves load-bearing performance, and a compact nutlet that houses the seed embryo. Embedded within the wing are vascular bundles that radiate outward toward the tip and curve downward toward the trailing edge. Notably, a cluster of thicker vascular tissue near the leading edge helps to create an optimal chordwise mass distribution, which plays a critical role in resisting bending and torsional deformation under aerodynamic load.

In planform, the wing often exhibits a conical geometry, with the maximum chord length occurring approximately two-thirds of the way from the base. Additionally, a protruding bulge near the seed base shifts the centre of mass off the rotational axis, introducing a deliberate mass asymmetry that enhances rotational stability. Microstructural surface features—especially roughened textures near the thickened inner span—are believed to contribute to aerodynamic performance by increasing torque around the rotational axis under low Reynolds number (Re ≤ 10^3^) conditions.^44–46^

One of the most distinctive characteristics of maple samaras is their inherent ability to autorotate upon release, regardless of their initial orientation or drop conditions^42^ (Fig. 2b). This self-stabilizing behaviour results from a finely balanced interplay between gravity, inertia, and aerodynamic forces.^47–49^ Beyond static force balance, the aerodynamic efficiency of samaras is critically enhanced by the generation of a stable leading-edge vortex (LEV) (Fig. 2c). This tornado-like vortex originates at the wing's leading edge and remains attached along the upper wing surface, reinforcing the circulation and generating a region of low pressure above the wing. The formation of the LEV is attributed to a spanwise transport of vorticity and a corresponding helical flow pattern that wraps along the wing's curvature. This coherent vortex structure generates a suction force on the leeward surface of the wing, significantly enhancing lift and rotational torque, which together sustain the autorotational descent. The LEV plays a dual aerodynamic role: it not only increases lift, especially under high angles of attack, but also stabilizes the local airflow, delaying flow separation and stall. While some studies attribute lift augmentation directly to the pressure reduction within the LEV core, others suggest that the LEV's primary function is to regulate the air stream over the wing, thereby ensuring consistent aerodynamic performance throughout the descent.^50–53^

In nature's repertoire of passive aerial innovations, the gliding seeds of *Alsomitra macrocarpa* represent another uniquely elegant solution for long-distance dispersal – one that defies conventional aerodynamic expectations^26,54^ (Fig. 2d). Unlike typical gliders that achieve efficiency through high aspect ratio wings, these seeds employ low aspect ratio, membrane-like wings yet exhibit remarkable flight stability and performance, even in the absence of gusts or sustained ind. Historically, their unusual shape has inspired early designs of manned gliders, highlighting their relevance not only to botany but also to the origins of aeronautical engineering. Despite their complex aerodynamic behaviour, they achieve stable flight without vertical stabilizers or active control systems.

Each *Alsomitra* seed features an ultra-light, paper-thin wing with a surface area disproportionately large relative to its mass. Its centre of gravity lies just ahead of the aerodynamic centre, a configuration that facilitates stable, forward gliding. The wing itself typically adopts a swept-back, tapered geometry with a reflexed trailing edge and finely tuned mass distribution. These design features collectively optimize lift generation while maintaining aerodynamic stability throughout the descent. Cross-sectional thickness is often less than 1 mm, further minimizing drag and enabling smooth airflow over the surface. *Alsomitra* seeds have been reported to exhibit a lift-to-drag ratio (L/D) of 3.7 and a lift coefficient (*C*_L_) of 0.34, with an average terminal descent velocity of just 0.41 m s^−1^.^55^ These values surpass those of autorotating seeds, which typically descend at approximately 1 m s^−1^, positioning *Alsomitra* among the most aerodynamically efficient passive flyers in the plant kingdom.

### 2.2 Pappus-bearing seeds

Among the diverse strategies evolved by flowering plants for seed dispersal, wind-mediated transport *via* filamentous structures – most notably seen in pappus seeds^43^ – represents another elegant passive aerial mechanisms. The dandelion (*Taraxacum officinale*) provides a striking example (Fig. 2e). Each seed is topped with a lightweight crown of fine bristles called a pappus,^56,57^ which enables extended suspension in the air and long-distance travel using even modest breezes. Beyond functioning as a simple parachute, the pappus maximizes aerodynamic drag, reduces terminal velocity, and stabilizes the seed's orientation during descent.^58,59^ The efficacy of this mechanism arises from the nuanced interaction between the porous pappus and the surrounding airflow, particularly in low Reynolds number regimes. Each filament within the pappus experiences a local Reynolds number defined as Re_f_ = *Ud*/*ν*, where *U* is relative flow speed, *d* is filament diameter, and *ν* is air's kinematic viscosity. At these small Re values, thick boundary layers form around each filament. These layers interfere with one another – a phenomenon known as the wall effect – which significantly reduces the airflow passing through the pappus, enhancing drag by reducing its effective permeability.^29,60–62^ This enhanced drag is not merely a result of increased surface area but arises from a unique aerodynamic phenomenon: the formation of a separated vortex ring (SVR)^29,63^ (Fig. 2f). As the seed falls, a toroidal vortex forms downstream of the pappus, creating a stable low-pressure region above the seed. This SVR stabilizes and slows descent, with drag – rather than lift – dominating the flight. The formation of the SVR critically depends on the porosity (*ϕ*) of the pappus. Experiments have shown a threshold value (ϕ* ≈ 0.93), beyond which a coherent, stable vortex wake emerges. Below this threshold, the wake breaks down, destabilizing the seed's flight path.^29,64,65^

The geometry of the pappus is also the key. One important variable is the opening angle (*α*) of the bristle array. A wider angle—approaching 180°—generates a higher drag coefficient (*C*_D_). Across Reynolds numbers ranging from 100 to 1000, a fully open pappus produces approximately 1.7 times more drag than one partially closed to 40°.^66–68^ Adding to this complexity, the dandelion's dispersal apparatus exhibits an environmentally responsive design. In humid conditions, hygroscopic tissues at the seed base swell, triggering the closure of the pappus (Fig. 2g). A specialized annular structure composed of vascular and cortical tissues provides the mechanical foundation for this movement. Differential swelling between the vascular and cortical layers drives the upward motion of the filaments, transitioning the pappus into a closed configuration. This transformation reduces drag, which offers two adaptive advantages: first, it helps prevent premature dispersal during humid or rainy conditions—when increased air density and typically lower wind speeds render long-distance travel inefficient—by enabling the seed to remain attached to the parent plant until dry, windy conditions return; second, it facilitates landing and anchoring in moist environments. Additionally, for the same projected area, the closed form yields lower unit drag than the open state, further streamlining the descent process. Through dynamic modulation of porosity, geometry, and flow interactions, dandelion seeds achieve remarkable control over passive flight.^69,70^ Their ability to remain airborne in flows while adapting to humidity exemplifies how biological structures elegantly balance form and function.

In addition to classic pappus-bearing seeds, poplar (*Populus* spp.) seeds employ a distinct wind-dispersal strategy, relying on dense tufts of fluffy, cotton-like fibers that enable them to remain aloft over substantial distances (Fig. 2h). These fine hairs increase drag and reduce terminal velocity, enhancing the seeds' residence time in the air and promoting long-range dispersal. Observations have documented dispersal distances exceeding 30 km under favorable wind conditions, particularly during storm events. It has been hypothesized that in turbulent weather, these seeds may be lofted above their original release altitude by convective air currents before descending gradually in a gliding manner.^71,72^ While the precise aerodynamic mechanisms governing this behaviour remain poorly characterized, the irregular, filamentous structure of the seed hairs likely plays a critical role in modulating drag and lift within turbulent flow regimes. The anisotropic and flexible nature of the fibers may also contribute to dynamic stabilization and prolong suspension. Though lacking the ordered porosity of canonical pappus structures such as those in dandelions, the poplar seed's stochastic architecture offers promising insights into collective dispersal strategies and may inspire alternative designs for passive aerial systems. Further study of their flight mechanics could enrich our understanding of turbulence-mediated seed transport and inform the development of fiber-based dispersal devices in synthetic systems.

Nature provides elegant solutions for energy-efficient flight through passive structures such as winged or parachuting seeds. However, these systems shape-morph slowly and lack real-time tunability. To engineer truly autonomous airborne robots, three key capabilities are essential: rapid shape-morphing through robotic materials, ultralight structural components, and remote energy delivery mechanisms that can operate over long distances. Light-actuated thin films potentially satisfy all three requirements, offering fast, reversible deformation, minimal mass, and precise control *via* collimated illumination. As such, they represent a promising foundation for untethered aerial robotics inspired by, but surpassing, natural paradigms.

## 3. Light actuated thin film for airborne robotics

Controlling aerial robots based on stimuli-responsive materials in mid-air requires efficient energy transmission over long distances. Conventional actuation methods – such as magnetic fields, electric fields, and chemical reactions – have demonstrated success in micro/nanoscale robotics but are fundamentally constrained by spatial attenuation or environmental limitations, rendering them unsuitable for wide-area applications.^73–77^ In contrast, optical stimuli, delivered *via* LEDs or lasers, provide a scalable and non-contact solution capable of inducing precise structural deformation from a distance.^78–80^ Laser beams, in particular, can be tightly collimated to target specific robots with high spatial selectivity, enabling control ranges that extend to hundreds of meters or more. This capacity for remote, directional energy delivery makes light a uniquely powerful tool for programming motion and behaviour in distributed airborne robots. Moreover, the ability to address multiple agents independently within a shared volume – by modulating beam position, intensity, or wavelength – introduces good flexibility for orchestrating complex collective tasks. These advantages position light as a superior actuation modality for scalable, untethered aerial robotics in both indoor and outdoor environments. In the following, we introduce a few robotic thin film materials capable of photomechanical shape-morphing.

### 3.1 Light driven liquid crystalline networks

**Fig. 3:**
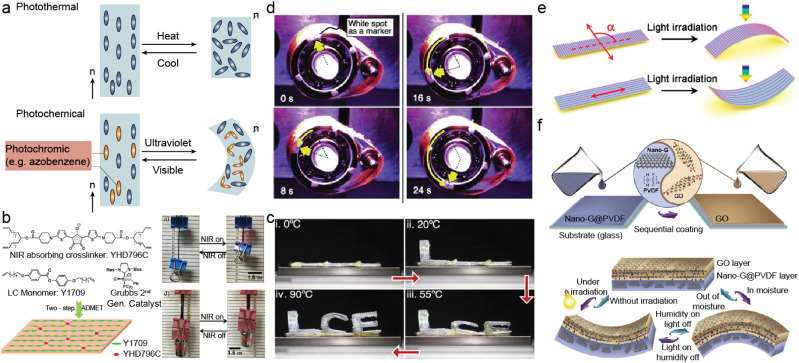
Light deformable polymer films. a) Schematic illustrations of LCN deformations *via* photothermal and photochemical mechanisms. b) Left: Molecular architecture of the LCN network; right: optical images showing an LCN ribbon before and after near-infrared (808 nm) irradiation, reproduced from ref. 94 with permission from ACS, copyright 2017. c) LCN material with an actuation temperature close and below ambient temperatures, reproduced from ref. 95 with permission from John Wiley & Sons, copyright 2022. d) Time-lapse images capturing the rotational motion of a light-driven plastic motor powered by an LCN laminated film under simultaneous ultraviolet and visible light at ambient temperature, reproduced from ref. 107 with permission from John Wiley & Sons, copyright 2008. e) Photo-bending responses of bimorph soft actuators with different carbon nanotube alignment directions, reproduced from ref. 113 with permission from ACS, copyright 2016. f) Schematics showing the fabrication process and actuation mechanism of a Nano-G@PVDF/GO bimorph films, reproduced from ref. 117 with permission from Elsevier, copyright 2020.

Liquid crystal networks (LCNs) are polymeric systems formed by crosslinking liquid crystalline monomers into a three-dimensional network. These materials retain the anisotropic molecular alignment of liquid crystals while gaining mechanical strength and responsiveness through the polymer network.^80,81^ The crosslinking process constrains the mobility of local liquid crystal (LC) domains, resulting in a mechanically anisotropic structure that can respond collectively to external stimuli. Upon exposure to elevated temperatures, this localized order can be disrupted, leading to a phase transition from ordered mesophases – such as the nematic state – to an isotropic phase. In aligned LCNs, this order-to-disorder transformation manifests as a macroscopic, anisotropic shape change, which is both elastic and reversible due to the entropic memory embedded in the polymer matrix.^82–85^ Depending on the crosslinking density, LCNs can be classified as lightly crosslinked liquid crystal elastomers (LCEs) or highly crosslinked glassy networks. In this work, we refer to both forms using the general term “LCN” unless otherwise specified. These materials have garnered considerable interest as stimuli-responsive actuators due to their capacity for large, reversible, and programmable shape transformations in response to diverse environmental stimuli.^86,87^ Photoactuation in LCNs occurs primarily *via* two mechanisms: photothermal and photochemical actuations (Fig. 3a).

#### Photothermal mechanism

In the photothermal route, light-absorbing agents embedded in the LCN matrix convert incident photons into localized heat, thereby triggering a phase transition from the nematic to the isotropic state and producing a contraction along the molecular alignment direction.^88–91^ This photothermal response is rooted in a dual thermomechanical interplay: the entropy-driven deformation of the amorphous polymer chains and the enthalpy-driven phase transition of the LC domains. Together, these effects enable rapid and reversible deformation upon light exposure. When doped with photothermal transducers – ranging from inorganic nanoparticles to organic dyes – or chemically modified with photoabsorptive moieties, LCN actuators can be remotely triggered with sub-second responsiveness.^92,93^ These additives act as nanoscale heaters, dissipating the energy *via* non-radiative relaxation, which induces localized temperature gradients that deform the LCN structure with high strain^94^ (Fig. 3b).

For typical LCN geometries used in soft robotic or microrobotic actuators about tens of micrometres in thickness and millimetres in lateral dimension, the full actuation cycle, encompassing heating and relaxation, occurs within sub second to several seconds. This response time is matched to the demands of aerial locomotion, particularly in microfliers operating under passive control schemes where rapid actuation is crucial for stability and steering in complex aerodynamic environments.

A further advantage of LCN-based actuators is their thermal tunability. By designing LC monomers with inherently lower phase transition temperatures and adjusting the crosslinking density, the onset of deformation can be brought within or even below ambient temperatures^95,96^ (Fig. 3c). This not only lowers the energy threshold required for activation, reducing the necessary light intensity, but also makes it possible for the system to respond to naturally available irradiance such as that of sunlight.^97,98^ For example, A liquid crystal elastomers crosslinked with Diels–Alder bonds and doped with multi-walled carbon nanotubes exhibit a low nematic-to-isotropic transition temperature, enabling self-sustained locomotion and deformation powered by ambient or body heat, or even natural sunlight.^99^ Furthermore, these actuators demonstrate versatile light-driven motility: under simulated sunlight (48 mW cm^−2^), they undergo horizontal self-rolling, while under natural sunlight, they perform topology-agnostic, sunlight-gated climbing on vertical surfaces of varying curvature and material. The possibility of achieving actuation at solar flux levels will open new frontiers for the deployment of untethered, energy-autonomous airborne robot in outdoor or large-scale scenarios.

#### Photochemical mechanism

Photoisomerization offers fundamentally distinct mechanism for actuation in light-responsive LCN. In contrast to photothermal pathways photoisomerization induces deformation through direct, light-triggered conformational changes at the molecular level.^100^ Among different photoswitch molecules, azobenzene stands as one of the most extensively studied and utilized chromophores.^101^ Its light-induced *trans*–*cis* isomerization has become the foundation for a wide range of responsive LCN systems.

Azobenzene exists in two thermally stable geometric isomers: a linear, rod-like *trans* configuration and a bent *cis* configuration. Upon ultraviolet (UV) irradiation, *trans*-azobenzene molecules absorb photons and undergo isomerization to the *cis* form. When azobenzene moieties are incorporated into the liquid crystalline framework, either as side chains or integrated into the polymer backbone, they serve as light-addressable units capable of disrupting local molecular order. Under UV light, the *trans*-to-*cis* transition alters the shape and polarity of the azobenzene, disturbing the mesogen alignment and inducing a local phase change from nematic to quasi-isotropic.^102^ This transformation results in anisotropic contraction along the mesogen alignment direction, often manifested as uniaxial bending toward the light source due to the light absorbing gradient. The process is fully reversible: exposure to visible light or gentle heating triggers the *cis*-to-*trans* reversion, restoring the original mesogen order and macroscopic shape.^103–106^ This bistable, light-controllable cycle enables repeatable and non-volatile actuation without requiring sustained energy input to maintain a given state.

A particularly striking example is the development of monodomain azobenzene-LCN films aligned along a predefined director axis. Under 360 nm UV illumination, these films exhibit light-induced bending toward the source, while exposure to 450 nm blue light restores the original flat configuration. Such precise control enables the fabrication of light-driven soft actuators with continuous and reversible deformation cycles. Laminating these LCN films onto a flexible polyethylene (PE) substrate further enhances their mechanical properties, allowing them to generate work output. For instance, a photoresponsive belt can continuously drive a motor upon constant dual light irradiation^107^ (Fig. 3d).

These capabilities open promising avenues for untethered aerial robotics, particularly at small scale. The bistability and directionality of azobenzene-LCN deformation allow for the design of deployable structures that remain static in one configuration and are activated to morph dynamically upon exposure to specific wavelengths. For example, a structure could be held in a folded configuration under UV light, released into the air, and then undergo rapid in-flight deformation triggered by visible light. This wavelength-selective control enables temporal and spatial decoupling of deployment and actuation, a feature that is especially valuable for airborne applications requiring delayed or mid-flight transformation.

### 3.2 Light-driven bilayer actuator

A light-driven bilayer soft actuator is a flexible bilayer structure composed of two physical or materials with distinct chemical properties, where at least one layer is responsive to optical stimuli.^108^ The essential operating principle of such actuators lies in the differential deformation between the active and passive layers, resulting in macroscopic curvature change. This deformation mismatch is achieved by integrating light-active agents – either photothermal converters or molecular photo-switches – into the active layer, enabling light-triggered actuation.^109–111^

In systems where photothermal nanomaterials are randomly dispersed within the active layer, uniform thermal expansion is typically observed upon illumination. This isotropic deformation induces straightforward bending toward the passive layer, often without introducing torsional components. However, by strategically orienting anisotropic nanomaterials such as aligned carbon nanotubes (CNTs), it becomes possible to engineer spatially programmable deformation profiles in response to directional light exposure.^112^ This anisotropy introduces a high degree of tunability, allowing the actuator to morph into more complex shapes. For instance, a paraffin-based coating containing aligned CNTs, laminated onto a polyimide (PI) substrate, demonstrates programmable shape morphing under visible light^113^ (Fig. 3e). By varying the cutting angle of the actuator strip relative to the CNT alignment direction, diverse bending modes—such as curling, twisting, or coiling—can be generated. These deformations can be triggered within milliseconds, enabling rapid response times. Moreover, such actuators have exhibited excellent fatigue resistance, maintaining mechanical performance after more than 100 000 actuation cycles.

Beyond thermal mechanisms, environmental responsiveness can be further amplified by exploiting the hygroscopic nature of functional nanomaterials. Many nanomaterials and polymer matrices feature abundant hydrophilic groups, allowing them to absorb water molecules efficiently. Under elevated temperatures or light-induced heating, these materials undergo reversible volume contraction due to desorption of previously absorbed water, enabling a secondary mode of actuation.^114–116^ One exemplary system utilizes a bilayer configuration of graphene oxide (GO)—which contains abundant oxygen-containing functional groups—and nano-graphite (Nano-G), both integrated with a polyvinylidene fluoride (PVDF) matrix^117^ (Fig. 3f). GO's high sensitivity to humidity stems from its numerous hydrophilic sites, which facilitate reversible water uptake and release. When exposed to either light or moisture, the GO layer swells or shrinks, generating biomorph actuation. This coupling of light and humidity responsiveness enables bidirectional actuation, offering multifunctional shape control in diverse environmental conditions.

### 3.3 Outlook for light driven actuator materials

While light offers a unique advantage for wireless, high-resolution manipulation – allowing precise control of remote microstructures with tunable wavelength selectivity – it also presents operational constraints. Active optical beam control typically requires a feedback-control loop and precise beam alignment. Although modern laser-scanning techniques may allow simultaneous control of multiple flyer units, concerns arise regarding operational complexity and laser safety.

For large-scale environmental monitoring, the discussed research takes a hybrid or semi-autonomous strategies, where light functions primarily as an environmental trigger rather than a continuous control of laser beams. By designing photomechanically preprogrammed materials, the flyers can self-adapt to light intensity or direction—transforming illumination into a guiding environmental cue rather than a direct actuation command. Along this line, the sunlight driven material can already offer a promising pathway for light tuned flyers. Several groups have already demonstrated sunlight-driven oscillators and actuators, underscoring the feasibility of leveraging ambient light for large-scale, low-cost deployment of airborne micro-robots.^97,98^

## 4. Light-actuated flyers

With nature offering a blueprint of aerodynamic elegance, mechanics now well understood,^43^ and responsive materials reaching new heights of performance, the question arises: how close are we to realizing human-controllable airborne flyers? Recent advances in light-responsive thin films capable of dynamic mid-air movements attempt to address this question.

### 4.1 Spinning flyers

**Fig. 4:**
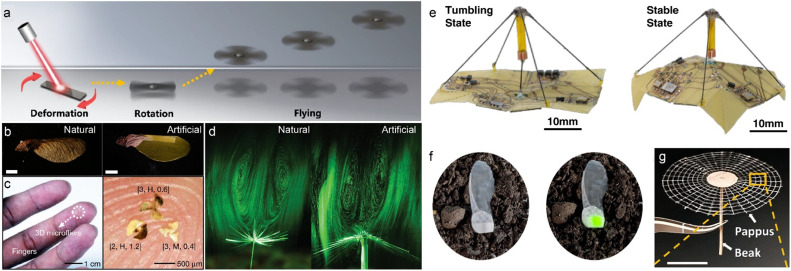
Light-actuated flyers. a) Schematic illustration of the light-induced rotary flyer taking off, reproduced from ref. 118 with permission from Springer Nature, copyright 2023. b) Photographs of a natural maple samara and a biomimetic artificial samara, reproduced from ref. 38 with permission from Springer Nature, copyright 2024. c) Photograph and optical micrograph of 3D microfliers, reproduced from ref. 40 with permission from Springer Nature, copyright 2021. d) Flow visualization of detached vortex ring structures generated by a natural dandelion diaspore and a synthetic polymer assembly, reproduced from ref. 33 with permission from John Wiley & Sons, copyright 2023. e) Origami microflier in its tumbling state (left) and stable descent state (right), reproduced from ref. 39 with permission from AAAS, copyright 2023. f) Fluorescent I-SeedSam resting on topsoil under ambient daylight (left) and daylight with additional excitation from a 980 nm laser pointer (right), reproduced from ref. 127 with permission from AAAS, copyright 2023. g) Photograph of an artificial *Tragopogon*-inspired structure, reproduced from ref. 125 with permission from John Wiley & Sons, copyright 2025.

As detailed in section 2, one of the most emblematic examples of passive flight in nature is the autorotating samara of the maple tree. These winged seeds descend in a helical trajectory, much like miniature helicopters, their flight path stabilized by inherent mass distribution and lift-enhancing rotational dynamics. Recently, this mechanism has been successfully translated into a light-actuated microscale rotor that can take off^118^ (Fig. 4a). This artificial flyer leverages the synergy between photothermal graphene and hygroscopic agar/silk composites to form a biomimetic propeller, which, when illuminated by near-infrared light, initiates rapid autorotation reaching angular velocities of up to 7200 rpm. Although mid-air manoeuvrability remains limited, this device represents the first known demonstration of liftoff in a responsive material system through propeller-based air propulsion aerodynamics. Another exemplar is an artificial winged seed capable of dynamic morphological transformation in the passive flight^38^ (Fig. 4b). This system employs azobenzene-functionalized LCNs, which undergo *trans*–*cis* photoisomerization upon UV-blue exposure. This molecular rearrangement causes the artificial wings to flatten mid-flight, reducing the terminal descent velocity from 98.4 cm s^−1^ to 88.2 cm s^−1^ and extending aerial hang time. The flight performance is continuously tunable: as the UV dose increases, both the descent speed (*V*_T_) and angular velocity (*Ω*) decrease in a controllable manner, enabling adjustments to flight trajectory in the mid-air.

To expand upon the passive flight paradigms established by maple, recent efforts have integrated three-dimensional microstructures to enhance both onboard sensing functionality and aerodynamic efficiency. Researchers have employed mechanically guided assembly to transform planar micropatterned films into helically folded 3D flight architectures^40^ (Fig. 4c). These structures exhibit tunable kinematic parameters, such as angular velocity, descent rate, and rotational stability. Importantly, scalable photolithographic fabrication enables mass production at micron and sub-millimeter resolutions.

### 4.2 Dandelion flyer

Unlike autorotating seeds such as those of maple, dandelion diaspores employ a drag-dominated parachute strategy, achieving slow and stable descent *via* a highly porous, filamentous crown. This resistance-based mechanism, governed by flow-induced vortex structures and low Reynolds number aerodynamics (Fig. 2f), offers a compelling blueprint for energy-efficient aerial robotics. Drawing inspiration from it, researchers have developed untethered, millimeter-scale passive flyers capable of controlled aerial descent by equipping with stimuli-responsive materials to dynamic modulate the aerodynamic drag. Light-responsive soft actuator strip made of LCN that can reversibly deform, opening or closing filament arrays (the synthetic pappus) under illumination^33^ (Fig. 4d). This mechanism enables real-time modulation of terminal velocity and descent trajectory, granting the flyers adaptive behaviour to ambient lighting – an ability akin to dandelion's humidity-triggered dispersal. Similarly, dandelion-inspired flight has been achieved using a light-driven bilayer actuator, in which the cutting angle of the ultralight actuator strip (polyimide–low-density polyethylene, ∼4 mg) imparts chirality during descent, thereby enriching and diversifying the aerodynamic behaviour.^34^ Beyond light, further efforts have integrated multimodal responsiveness into such artificial diaspores. By combining MXene with polyethylene bilayers, researchers fabricated actuators that respond not only to optical cues but also to humidity, temperature, voltage, and volatile solvents.^119^ For example, differences in thermal expansion between the polyethylene and MXene layers induce bending under thermal, optical, or electrical stimulation, while their contrasting sensitivities to water and solvents drive curvature in humid environments. These coupled deformations can be transduced into electrical signals, effectively converting humidity, infrared light, and temperature inputs into readable outputs. Such multimodal responsiveness greatly broadens the actuator's functional scope in soft robotics. These diverse triggers also expand the range of achievable flight behaviours, offering tunable control over descent speed, trajectory, and stability, without onboard electric power computation.

### 4.3 Parachute typed flyer

Parachute-type structure provides a simple yet highly effective mechanism for controlled descent at small scales. By harnessing aerodynamic drag, they enable soft landing without the need for active control systems, reducing both energy consumption and structural complexity. Researchers introduced a battery-free origami-inspired microflier that actively alters its aerodynamic configuration in midair^39^ (Fig. 4e) through the implementation of a low-power electromagnetic actuator. The actuator produces peak forces of up to 200 millinewtons in just 25 ms, allowing a quick morphing toggling between two configurations in flier winglets: an expanded state that induces tumbling descent for increased lateral displacement, and a folded state optimized for stable vertical descent with minimal drift. The electromagnetic actuator and onboard electronics are powered entirely by ambient light energy *via* onboard solar cell panels. This system presents a pinnacle example for the convergence of origami engineering, low-power actuation, and autonomous energy harvesting in a micro sized electronic aerial robot. In our perspective, the ultimate development goal of responsive thin-film flyers is to replicate the sophisticated functionalities of electronics, but with greater emphasis on energy efficiency (*e.g.*, harnessing wind power), adaptability (self-response to environmental conditions), and biological sustainability through the use of biodegradable materials.

### 4.4 The onboard functions

Stimuli-responsive polymer thin film and other actuator materials have enabled flyers to dynamically tune their aerodynamic properties, allowing motion to be actively modulated by external cues such as light, humidity or heat. To further enhance the functionality of these flight platforms, towards the application of distributed sensing and environmental monitoring, attempts to integrate microscale sensors have been done.

The 414 milligram microfliers described above integrate a compact suite of electronics, including programmable microcontrollers, Bluetooth transmitters, and environmental sensors, allowing them to autonomously collect and wirelessly transmit data over distances up to 60 meters. Even under gentle breezes, these devices can traverse nearly 100 meters, highlighting the potential of ultra-lightweight platforms for distributed aerial sensing.^39^ Similar strategies have incorporated ultraviolet sensors and battery-free wireless modules capable of capturing multiple environmental parameters, such as pressure, humidity, temperature, and irradiance.^31,40^

The pursuit of environmentally sustainable sensing strategies has motivated a departure from conventional electronic devices and stimulated the exploration of material-intrinsic sensing approaches. The change of the dandelion morphology is proposed to reflect the local information such as humidity^70,120,121^ (Fig. 2g). The hygroscopic deformation of the pappus, which passively modulates drag as a function of moisture, represents a naturally informed strategy of responsive dispersal. Such built-in responsiveness is inherently suited for stimuli-responsive flyers, where the structural design itself embodies the sensing function.^122^ Recent advances have extended this concept beyond distributed seeds. For instance, thin wooden veneers have been transformed into stiff, biodegradable humidity-driven actuators and further engineered into three-tailed seed carriers capable of autonomous self-burial under variable soil moisture.^123^ Similarly, artificial seeds exploiting humidity-driven mechanical effects have been reported, where angular displacement and diameter changes directly correlate with relative humidity variations across 30–90%, providing a quantitative link between environmental cues and morphological adaptation.^124^

Beyond mechanical shape change, chromatic transitions offer a visually intuitive route to environmental readout. By integrating colorimetric materials into dispersible flyers, local information – such as humidity, ultraviolet exposure, or pH – can be detected through direct optical inspection.^40,125,126^ More advanced approaches employ luminescent materials that emit in the near-infrared, a wavelength regime safe for human eyes and suitable for long-range optical interrogation^127^ (Fig. 4f). Such systems allow distributed flyers to serve as airborne, deployable sensors capable of relaying thermal or chemical information without embedded electronics.

This vision of material-based sensitivity fundamentally redefines environmental monitoring at small scales.^27^ Instead of relying on electronic circuits or wireless communication, the flyers themselves become the sensor. After deployment, information can be harvested non-invasively by drones or ground-based instruments, through colorimetric analysis of captured images or spectroscopic characterization of emission signals.^36^ This paradigm demonstrates how responsive matter can transform passive flyers into distributed, eco-compatible sensor networks, achieving real-time and remote monitoring under field conditions while minimizing ecological footprint.

## 5. Discussion

Conventional electrically actuated microflyers – such as those employing dielectric elastomer or piezoelectric actuators – can deliver high-frequency operation, yet their reliance on high-voltage supplies, rigid electrodes, and tethered connections significantly restricts miniaturization and environmental compatibility. In contrast, the light-driven microflyers presented in this review operate through passive flight mechanisms, achieving long-distance mobility powered solely by environment forces (*e.g.* gravity and wind drag). In terms of energy efficiency, the passive light actuated flyer exhibits a cost of transport (COT) of approximately 0.2 J kg^−1^ m^−1^ over distances of 10^5^–10^6^ meters, whereas conventional piezoelectric-based flight robots report a COT as high as 131 J kg^−1^ m^−1^ over distances around 10^3^ meters.^128^ Light in these systems also serves offers high spatial selectivity and easy integration into flexible architectures, enabling dynamic switching between flight modes such as takeoff, landing and gliding in certain directions. Since the actuation source (light) is externally, wirelessly available, and spatially distributed, it renders the proposed approach inherently energy-efficient and particularly suited for distributed environmental monitoring, in which low energy consumption and material sustainability often outweigh the need for precise control of the flight. Moreover, the stimuli-responsive nature of the light-driven polymer enables an intelligent response during flight. For example, one can design a flyer that opens its wings to glide during the daytime and closes its structure to land in the dark. The same principle can be applied to humidity-gated takeoff and landing in flyers equipped with humidity-sensitive arms. Conventional flyers often require electronic sensors and actuators to detect environmental conditions. In contrast, in responsive-polymer-based flyers, environmental sensing is intrinsically built into the material itself.

In parallel with the pursuit of new functionalities, environmental sustainability has emerged as one of the most critical considerations in the development of wind-dispersed aerial drones. This concern stems from several realities: large-scale production, the practical impossibility of retrieving all devices after deployment, and their inevitable interaction with ecosystems – ranging from soil and plants to birds and insects. Thus, sustainability cannot be treated as an afterthought but must be embedded as an ethical imperative in the technology's design pipeline, with ecological compatibility achieved before any wide-scale implementation.

One promising strategy has been the adoption of biodegradable structural materials.^129^ For instance, porous cellulose acetate, a bio-derived polymer, has been employed to fabricate passive microfliers^126^ (Fig. 4g). These ultralight structures retain aerodynamic performance while degrading harmlessly after release, thereby minimizing ecological footprint. In a related advance, biodegradable polylactic acid was combined with lanthanide-doped phosphors to create a bioinspired, biocompatible luminescent artificial seed.^128^ Such examples illustrate a viable route toward scalable, eco-friendly deployment of aerial sensors that blend seamlessly with natural cycles of degradation.

However, complete biodegradability – including electronic and sensing components – remains a big challenge. Fully biodegradable systems must balance mechanical integrity, environmental stability, and decomposition rate. For instance, a functional lifetime of days to weeks is typically desirable for environmental monitoring, ensuring sufficient flight endurance and data collection before degradation begins. The degradation process should produce non-toxic, naturally assimilable byproducts, minimizing risks to flora and fauna. Similarly, the total mass density of deployed microfliers must be kept below ecological thresholds (*e.g.*, <10 g m^−2^) to prevent accumulation or ingestion hazards. Emerging strategies are addressing these limitations through transient electronics, dissolvable metal circuits (Mg, Zn), and bio-compatible photonic sensors, enabling fully transient devices that vanish after mission completion. While we are still several years away from achieving complete biodegradability with integrated functionality, ongoing research in green materials chemistry and soft transient electronics is closing this gap rapidly.

The convergence of stimuli-responsive materials with passive flight principles is rapidly transforming the capabilities of small-scale aerial systems. Fast-responding actuators now allow in-flight modulation of aerodynamic properties, enabling untethered flyers to adapt dynamically to changing light, humidity, or temperature. This capacity for autonomous, low-energy adjustment represents a critical step toward self-regulating, energy-efficient robotic flight. As functionality extends to include sensing and data transmission, these miniature platforms are poised to play an important role in distributed environmental monitoring networks.

Yet, despite these advances, the field is only beginning to tackle the real challenges of airborne manoeuvrability and sensing. Moving forward, several key challenges must be addressed. First, the responsiveness and actuation efficiency of smart materials must be improved to allow reliable control over aerodynamic behaviour, even amid stochastic wind fluctuations. Second, multifunctionality must be integrated – embedding sensors, actuator systems, and computation – without sacrificing flight performance. Three complementary strategies are expected: first approach will focus on scaling up flyer size to accommodate more sensor elements; another will pursue scaling down to deploy swarms, which will necessitates the creation of miniaturized sensing and communication methods; a third pathway will explore the direct embedding of sensing or optical response into structural materials—for instance, through the use of mechanochromic or photoluminescent polymers. Although the degree of functionality achievable will still be constrained by weight limits and fabrication precision, this materials-centric strategy could offer a scalable and sustainable route to building highly efficient, multifunctional micro-aerial systems.

Finally, as airborne devices proliferate, material composition raises pressing ecological concerns, particularly regarding microplastic pollution. The next frontier in responsible design will require integrating biodegradable and eco-benign materials, ensuring that advances in bioinspired aerial robotics remain in harmony with the ecosystems they are ultimately designed to monitor. We hope this future will arrive soon.

Looking forward, bioinspired microfliers have the potential to occupy a distinct niche in distributed, passive, and environmentally sustainable sensing. Rather than competing with traditional drones, these future systems could operate in places where conventional aerial robots remain impractical – dense forest canopies, disaster zones, or fragile ecosystems where retrieval, continuous power supply, or active control are infeasible. If made ultralight and biodegradable, such microfliers could one day be deployed in large numbers without leaving a lasting ecological footprint, functioning as transient, disposable sensors that naturally degrade after completing their tasks. Embedding simple sensing functionalities directly into the material – such as colorimetric or luminescent indicators for humidity, UV exposure, or chemical cues – may enable data collection without batteries or on-board electronics. In this envisioned scenario, information could be retrieved remotely through aerial imaging or spectroscopic readout, allowing large-scale environmental monitoring with minimal maintenance and energy consumption.

## Author contributions

Jianfeng Yang, Hao Zeng: conceptualization, investigation, visualization, writing, review & editing.

## Conflicts of interest

There are no conflicts to declare.

## Data Availability

No primary research results, software or code have been included, and no new data were generated or analysed as part of this review.
